# Pyrazoline Hybrids as Promising Anticancer Agents: An Up-to-Date Overview

**DOI:** 10.3390/ijms21155507

**Published:** 2020-07-31

**Authors:** Dimitris Matiadis, Marina Sagnou

**Affiliations:** National Center for Scientific Research “Demokritos”, Institute of Biosciences & Applications, 153 10 Athens, Greece; sagnou@bio.demokritos.gr

**Keywords:** pyrazolines, hybrid compounds, conjugates, anticancer, antitumor, cytotoxicity, nitrogen heterocycles

## Abstract

Pyrazolines are five-membered heterocycles possessing two adjacent nitrogens. They have attracted significant attention from organic and medicinal chemists due to their potent biological activities and the numerous possibilities for structural diversification. In the last decade, they have been intensively studied as targets for potential anticancer therapeutics, producing a steady yearly rise in the number of published research articles. Many pyrazoline derivatives have shown remarkable cytotoxic activities in the form of heterocyclic or non-heterocyclic based hybrids, such as with coumarins, triazoles, and steroids. The enormous amount of related literature in the last 5 years prompted us to collect all these published data from screening against cancer cell lines, or protein targets like EGFR and structure activity relationship studies. Therefore, in the present review, a comprehensive account of the compounds containing the pyrazoline nucleus will be provided. The chemical groups and the structural modifications responsible for the activity will be highlighted. Moreover, emphasis will be given on recent examples from the literature and on the work of research groups that have played a key role in the development of this field.

## 1. Introduction, Overview, and Classification

Cancer is one of the leading causes of death worldwide. According to the World Health Organization reports, about one in six deaths globally is attributed to cancer with more than 9 million deaths in 2018 [1]. In simple terms, cancer is a large group of diseases in which abnormal cells divide without control and can invade nearby tissues. The main treatments include surgery, chemotherapy, and radiotherapy. Yet, there is no cancer treatment that is 100% effective for most tumor types. Drug-resistant cancers, low specificity, and high toxicity of chemotherapeutic agents resulting in serious side effects create an urgent need to develop new anticancer agents from novel scaffolds.

**Figure 1:**
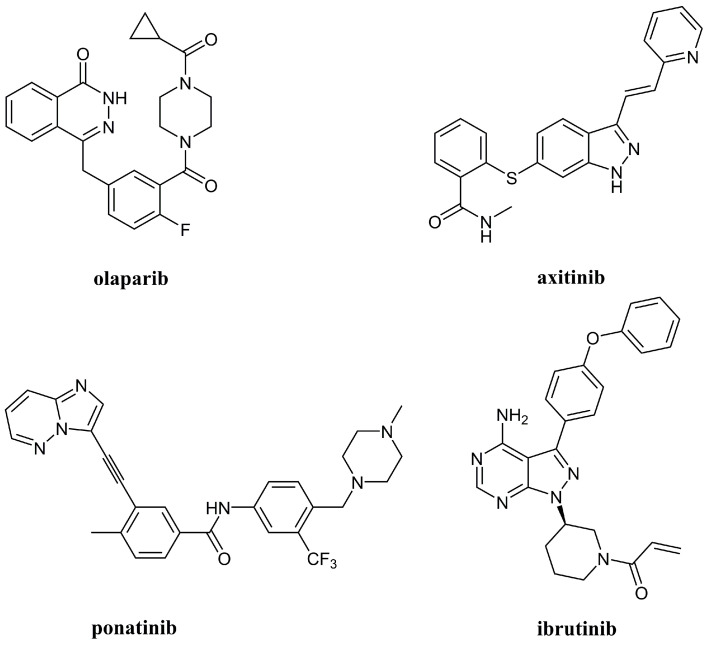
Nitrogen-nitrogen (N-N) heterocyclic anticancer agents that have received approval by FDA in the last decade.

A significant number of nitrogen heterocycles have been approved by the FDA as chemotherapeutic drugs in recent years [2]. Many of them incorporate a nitrogen-nitrogen (N-N) bond such as olaparib for the treatment of advanced ovarian cancer [3], axitinib that has received approval for renal cell carcinoma treatment [4], ponatinib for the treatment of chronic myeloid leukemia and Philadelphia chromosome-positive acute lymphoblastic leukemia [4], and ibrutinib as a first line treatment for chronic lymphocytic leukemia [5] (Figure 1).

In the recent years, a number of review articles for—or including—pyrazolines have been published. They are concerned with the structure, with emphasis on the synthetic strategies [6,7], with their biological activities in general [8,9,10,11], or they even focus on their interesting photophysical properties [12]. In fact, a few of them include pyrazoline as a substructure of pyrazole. Furthermore, it has been noticed that only one review exists on the therapeutic potential of steroidal pyrazolines [13], but there are none regarding the pyrazoline-hybrids in total and/or their anticancer activities.

Taking into consideration the fact that the research on this bioactive heterocycle as an anticancer agent is attracting increasingly more interest we decided to publish the present review to provide a comprehensive account of the cytotoxic pyrazoline conjugates in the recent literature.

**Figure 2:**
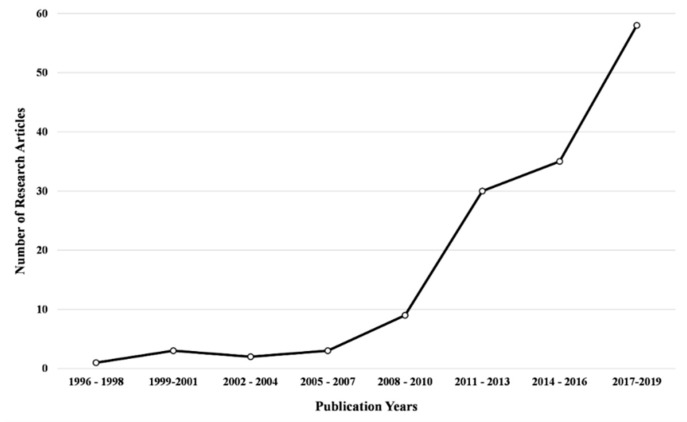
The number of research articles in pyrazolines in the anticancer activities field, excluding reviews and patents throughout the first appearance of a cytotoxic pyrazoline until 2019.

So far, over 150 research articles have been published reporting results on novel or known pyrazoline structures as potential anticancer agents including the design strategies, synthesis, *in vitro* and *in vivo* screening as well as investigation of the structure activity relationships or mechanistic insights. Most of them are relatively recent, up to 10 years old (Figure 2). The vast majority of the biologically active pyrazolines are the 2-pyrazoline isomers.

As a general trend, most of the published papers are related to pyrazoline hybrids, since molecular hybridization is an effective strategy for rational design of multitargeting agents incorporating two or more pharmacophores in one molecule [14].

We approached the field of 2-pyrazoline conjugates classifying them by their accompanying bioactive units. Simple and common heteroaromatic units like pyridine, furan, thiophene, attached to the side carbons or to nitrogen-1 of the ring and derived mostly from chalcones are not considered to form a hybrid and thus not included in the present review. Moreover, the structurally relevant pyrazolones (or pyrazolinones) are out of the scope of the present review as is any detailed discussion of the mechanism of biological activity of the conjugates referred herein

Pyrazolines, also referred to as dihydropyrazoles, constitute a class of five-membered heterocycles containing two adjacent nitrogens. Three types of pyrazolines exist depending on the site of the endocyclic double bond; 1-pyrazolines, 2-pyrazolines, and 3-pyrazolines.

Consequently, and resulting from the chemical classification of pyrazolines, the following sections will be covered:

- 1-Pyrazolines;
- 3-Pyrazolines;
- 2-Pyrazolines.
  ○ Clinical drug-pyrazoline hybrids.
  ○ Coumarin, quinoline, and other benzene-fused six-membered heterocyclic pyrazoline hybrids.
    ▪ Coumarin-pyrazoline hybrids.
    ▪ Quinoline and quinolinone pyrazoline hybrids.
    ▪ Other benzene-fused six membered heterocyclic pyrazoline hybrids.
  ○ Indole-pyrazoline hybrids and other benzene-fused five-membered heterocyclic pyrazoline hybrids.
    ▪ Indole-pyrazoline hybrids.
    ▪ Isatin-pyrazoline hybrids.
    ▪ Other benzene-fused five-membered heterocyclic pyrazoline hybrids.
  ○ Thiazole and thiazolidinone pyrazoline hybrids.
    ▪ Thiazole and benzothiazole pyrazoline hybrids.
    ▪ Thiazolidinone-pyrazoline hybrids.
  ○ Two nitrogen containing heterocyclic–pyrazoline hybrids and analogues.
    ▪ Pyrazole-pyrazoline hybrids.
    ▪ Imidazole and benzimidazole-pyrazoline hybrids.
    ▪ Other two heteroatom heterocyclic pyrazoline hybrids.
  ○ Three or more nitrogen heterocyclic–pyrazoline hybrids and analogues.
    ▪ Triazole-pyrazoline hybrids.
    ▪ Triazine-pyrazoline hybrids.
    ▪ Other three or more heteroatom heterocyclic-pyrazoline hybrids.
  ○ Steroidal pyrazoline hybrids.
  ○ Other non-classified pyrazoline based hybrids.

## 2. Literature Survey

The literature study was carried out up to April 2020 using reliable and well-established databases, namely Scopus, Google Scholar, and Reaxys. Keyword combinations such as pyrazoline(s), anticancer, antitumor, and cytotoxic were used. The first appearance of a cytotoxic pyrazoline in the literature was the synthesis of a taxol-pyrazoline conjugate in 1996 by Appendino, Jakupovic et al. [15]. Since then, a steadily grown research interest in this field shows the potential of this heterocyclic motif.

An indicative statistical analysis of the number of research articles, excluding the reviews, using the terms “pyrazoline” or “pyrazoline” and “anticancer” through Scopus, from 1996 until 2019, illustrates the abovementioned trend (Figure 2). The time period was divided into three-year intervals (1996–1998, 1999–2001, 2002–2004, 2005–2007, 2008–2010, 2011–2013, 2014–2016, and 2017–2019). Noteworthy, in between the two more recent intervals, the number of research articles has almost doubled (35 from 2014–2016 to 68 from 2017–2019).

Particularly, regarding the pyrazoline hybrids, it is worth mentioning that the scientific articles from 2013 until now represent more than 75% of the total papers for these hybrids.

## 3. 1-Pyrazolines

**Figure 3:**
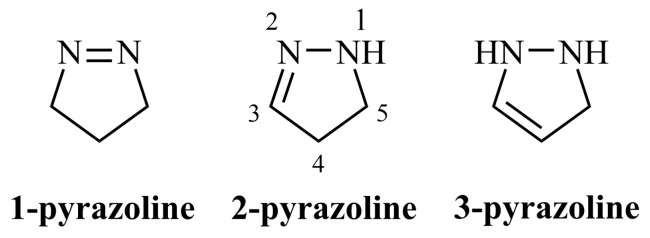
Core structure of pyrazoline ring along with the numbering of the biologically relevant 2-pyrazoline according to common nomenclature.

1-Pyrazolines are the isomers incorporating a nitrogen-nitrogen double bond (Figure 3). They are commonly synthesized by cycloaddition reaction between a diazoalkane and a cyclic or an acyclic alkene [16,17]. They are useful intermediates even for the synthesis of complex natural products [18] as they are converted to cyclopropanes or other structures by photolysis [17,19,20,21] or thermolysis [22,23,24]. The reported biological applications for this pyrazoline form are scarce. Citreoazopyrone is the only natural product containing a 1-pyrazoline ring having inhibitory activity against hypocotyls of lettuce seedlings [25].

**Figure 4:**
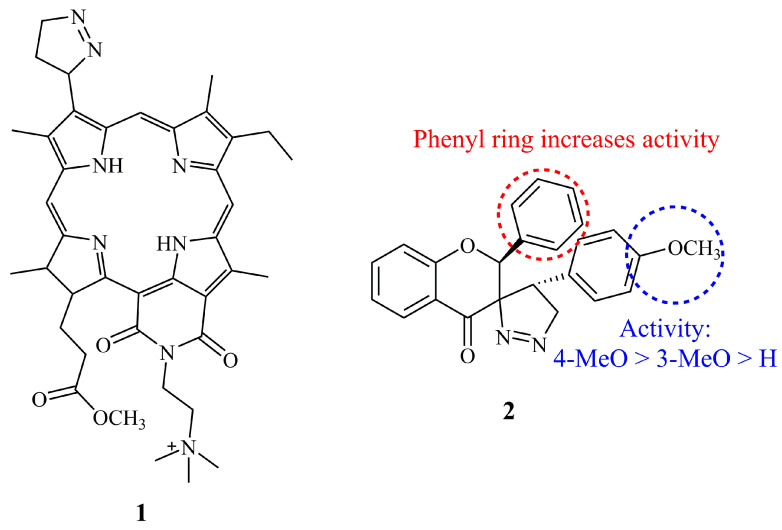
Chemical structures of anticancer 1-pyrazolines.

To the best of our knowledge only two reports exist for their anticancer activity. Sim et al. reported [26] the use of purinimide-pyrazoline compounds (e.g., derivative **1**) (Figure 4) and their polyoxometalate complexes as photosensitizers in photodynamic therapy. Recently, Budziz and co-workers documented the biological evaluation of a series of chromanone-spiro-1-pyrazoline hybrids [27]. Among them, **2** was found to be the most cytotoxic against HL-60, NALM-6, and WM-115 cell lines (IC_50_ = 3.0–6.8 μM). The presence of a *para*-methoxyphenyl group (Figure 4) proved essential since the non-substituted compound exhibited more than 330-fold lower cytotoxicity against HL-60. **2** induced 60% of HL-60 cells to be arrested in G_2_/M phase.

## 4. 3-Pyrazolines

Similarly to 1-pyrazolines, 3-pyrazoline isomers are under-represented in this review since the literature on their biological activities is limited. They are formed by cycloaddition or iodocyclization reactions [28] usually mediated by metal catalysts [29,30,31].

**Figure 5:**
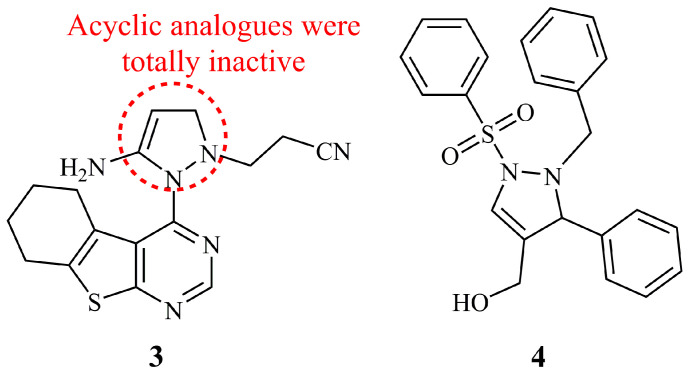
Chemical structures of anticancer 3-pyrazolines.

Among a library of thieno [2,3-d]pyrimidine derivatives, the hybrid 3-pyrazoline **3** was found as one of the most active, showing selective cytotoxicity against HepG_2_ and MCF-7 (IC_50_ = 3.96–4.38 μM). Since **3** was the only pyrazoline in that publication, no conclusions can be drawn on the SAR. However, the acyclic hydrazine intermediate analogue was inactive within the tested concentration range (IC_50_ values > 100 μM). As documented, **3** could arrest cell cycle, induce apoptosis, and serve as cytostatic agent by inhibiting/reducing Topoisomerase II [32]. Mamedova et al. studied the biological evaluation of pyrazine, pyridazine, and 3-pyrazoline sulphonilimine compounds with limited data on their anticancer activity [33]. Compound **4** was found active against MCF-7 but not against PC-3 cells (Figure 5). IC_50_ values were not reported. This report is mentioned herein parenthetically, since it is the second of the anticancer 3-pyrazoline libraries, though not hybrid.

## 5. 2-Pyrazolines

Among the three isomers of pyrazolines, 2-pyrazoline is the most common in literature with various applications as materials; brightening agents of synthetic fibers [34], fluorescent probes [35], metal ion recognition [36], hole-transport materials [37], optoelectronics [38], and as biologically active compounds with anti-inflammatory [39,40], antimicrobial [41,42,43], antiviral [44], antimalarial [43,45], trypanocidal [46], insecticidal [47], and anticancer properties. In this form of pyrazoline, the endocyclic double bond exists between the N-2 and the C-3.

### 5.1. Clinical Drug-Pyrazoline Hybrids

**Figure 6:**
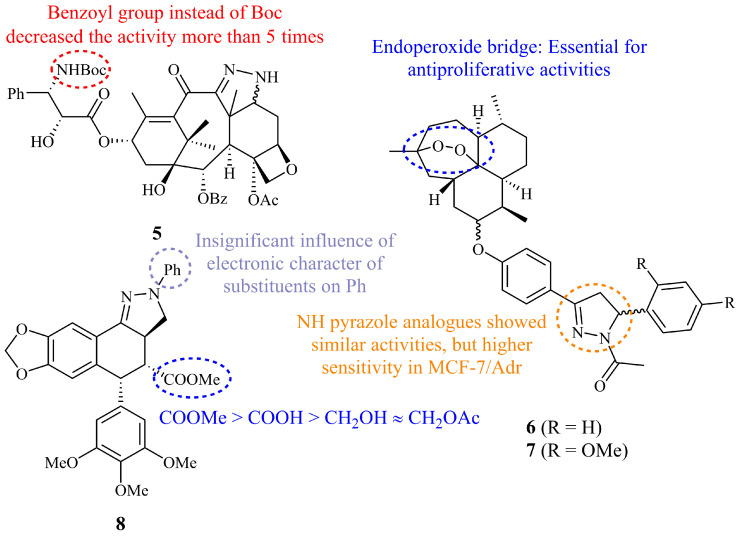
Chemical structures of anticancer clinical drug-pyrazoline hybrids.

The first report of pyrazoline anticancer drugs included the synthesis and cytotoxicity evaluation of Taxol (paclitaxel)–pyrazoline hybrids [15]. Six compounds incorporating an unsubstituted (NH) pyrazoline ring were prepared and their preliminary *in vitro* tests on MDA-MB-231 cells showed for molecule **5** (Figure 6) an IC_50_ value of 1.4 nM which was comparable to those of the positive controls, i.e docetaxel, paclitaxel, and *N*-debenzoyl-*N*-Boc-3′-dephenyl-3′-isobutyltaxol (IC_50_ values of 0.8, 2.4, and 4.7 nM, respectively).

Recently, 20 artemisinin-pyrazoline and pyrazole hybrids were synthesized and assessed for their anticancer activity by Zhao et al. [48]. Among them, compound **6** (*N*-acetyl pyrazoline) displayed the lowest GI_50_ values overall for HL-60, U937, K562, NB-4, PC-3, LNCaP, MDA-MB-231, MCF-7, and MCF-7/Adr tumor cell lines (GI_50_ = 0.025–0.42 μM), significantly lower than the parent dihydroartemisinin (GI_50_ = 0.15–33.22 μM). Interestingly, **6** and other tested analogues (Figure 6) showed unique sensitivity against MCF-7/Adr (Adriamycin-resistant cell line) over MCF-7 (for compound **6**, 2.5-fold higher potency and for compound **7**, 20-fold), indicating that these derivatives have potential to be developed as therapeutic agents to treat drug-resistant breast cancer.

Gordaliza group has reported the cytotoxic activities of many podophyllotoxin conjugates including oximes, imines, isoxazolines, pyrazoles, and pyrazolines [49]. Among these cytotoxic fused pyrazolignans, pyrazoline **8** (*N*-phenyl derivative) displayed the lowest IC_50_ values ranging from 1.0 to 1.9 μM for P-388, A-549, and HT-29 cell lines [50,51]. However, even though the IC_50_ values were at micromolar levels, the activity was over two orders of magnitude lower than this of podophyllotoxin, confirming that the lactone moiety of the latter is a prominent requirement for high antitumor activity.

### 5.2. Coumarin, Quinoline, and Other Benzene-Fused Six-Membered Heterocyclic Pyrazoline Hybrids

#### 5.2.1. Coumarin-Pyrazoline Hybrids

Coumarins are fused heterocycles, widely found in natural products. Their synthesis and evaluation of biological activities have attracted considerable attention from both organic and medicinal chemists. They exhibit potent anticancer activity, usually accompanied with low toxicity [52]. Moreover, they are convenient structures for drug development, since they have many sites for functionalization.

**Figure 7:**
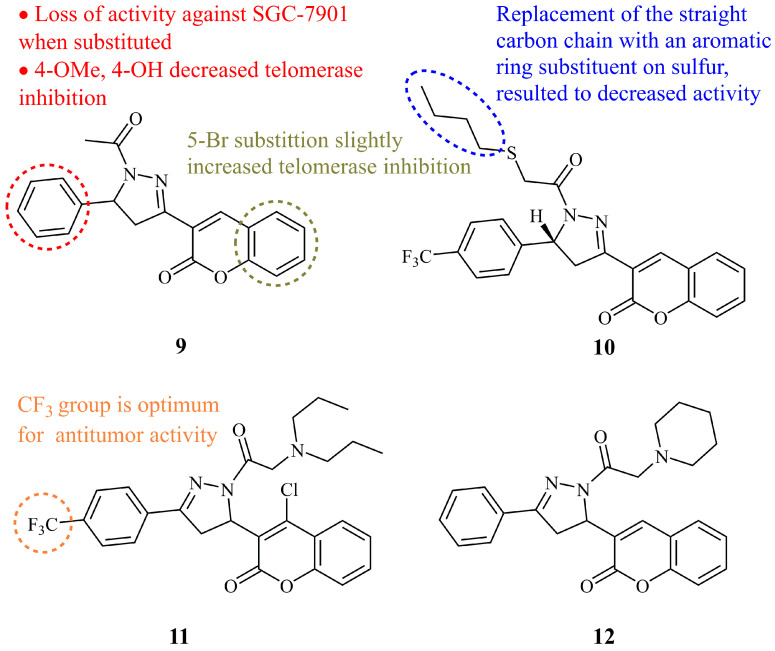
Chemical structures of anticancer coumarin-pyrazoline [55,56].

Over the last decade, many coumarin-pyrazoline hybrids have been synthesized and studied as cytotoxic agents. Liu and co-workers first synthesized seven simple molecules of this type and found that **9** (Figure 7) was the most active against human gastric cancer cell line SGC-7901 (IC_50_ = 2.69 μg/mL). In addition, compound **9** could strongly inhibit telomerase with IC_50_ value of 2.0 μM, while the chalcone precursor was found to be inactive within the tested concentration range. All the synthesized compounds exhibited poor activity against PC-3 and A431 cell lines [53]. Next, they reported a series of modified hybrids at the N-1 and C-5 substituents of the pyrazoline ring, incorporating a thioethanone group instead of acetyl, as potential telomerase inhibitors. Compound **10** (Figure 7) showed the most potent inhibitory activity against telomerase with IC_50_ value of 0.92 μM, exhibiting a moderate activity against SGC-7901, MGC-803, Bcap-37, and HEPG_2_ cell lines (IC_50_ = 2.35–8.68 μM). In general, the results suggested good correlation between antiproliferative and telomerase activity, indicating that inhibition of the telomerase by the active compounds caused inhibition of cancer cell growth. Compound **10** could suppress cell proliferation through inducing cell cycle arrest in G0/G1 phase [54]. In the same year, they reported another series of novel coumarin-pyrazoline hybrids concluding with compound **11** (Figure 7), which showed substantial increase in activity against HepG_2_ cell lines (IC_50_ = 1.12 μM), with similar telomerase inhibitory activity (IC_50_ = 0.98 μM). However, **11** displayed an increase of IC_50_ values for SGC-7901, MGC-803, Bcap-37 cell lines in comparison with the compound **10** by a factor of 2–3 [55]. More recently, the same group expanded their investigations on these hybrids by discovering the novel compound **12** (Figure 7) which was found to have high selectivity against tumor cells (IC_50_ = 1.41 μM for MGC-803) versus human non-cancerous cells (IC_50_ = 2.3 and 2.5 mM for GES-1 and L02, respectively). Studies on the mechanisms of action were also reported [56].

**Figure 8:**
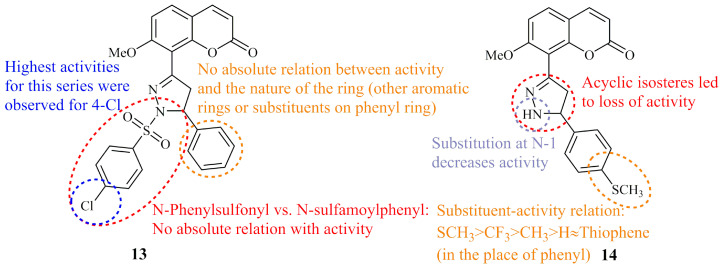
Chemical structures of anticancer coumarin-pyrazoline hybrids [57,58] Amin et al.

Amin et al. synthesized two groups of phenylsulfonyl and sulfamoylphenyl coumarin based pyrazoline hybrids. Selected compounds among the 24 molecules were screened by the National Cancer Institute (NCI) for their anticancer activity and compound **13**, bearing a *para*-chloride substituted *N*-phenylsulfonyl group, displayed the most potent selective cytotoxicity against MCF-7 and HCT-116 with GI_50_ values of 0.49 and 0.94 μM, respectively. Subsequently, the authors proceeded to screen all derivatives against human colon cancer cell line HCT-116. Most of them showed lower IC_50_ values than the control doxorubicin (IC_50_ = 0.63 μM) whereas for compound **13** (Figure 8) in particular, IC_50_ value was found at the range of 0.01 μM [57]. Two years later, the same group reported the anticancer activity against HepG_2_ cells of three more groups of coumarin bearing functionalized pyrazoline moieties comprising of unsubstituted (NH), *N*-phenyl, *N*-acetyl, and *N*-carbamide (urea-like) groups at N-1 of the ring. All compounds, including five non-cyclic isosteres that were synthesized for comparative reasons, were active at the nanomolar range with IC_50_ values under 100 nM. Compound **14** (Figure 8), incorporating a *para*-methylthiophenyl group at C-3 of the ring, was the most potent (IC_50_ = 10 nM) followed by the thienyl analogue (IC_50_ = 11 nM). Both of these derivatives were N-1 unsubstituted. Overall, the structure activity relationships suggested that the activity was influenced by the substitution pattern on pyrazoline ring [58].

**Figure 9:**
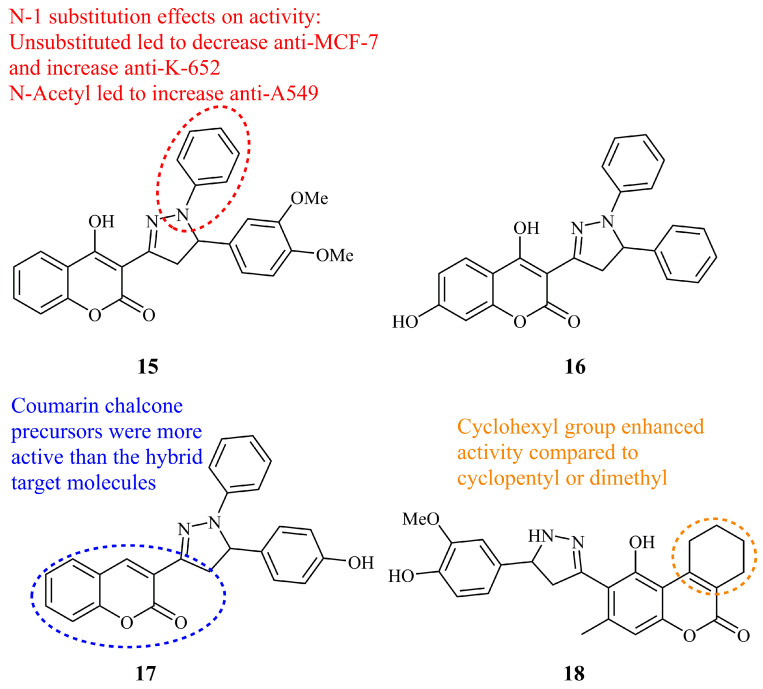
Chemical structures of anticancer coumarin-pyrazolines.

4-Hydroxycoumarin derivatives with unsubstituted (NH), *N*-phenyl and *N*-acetyl pyrazoline hybrids were prepared by Latif et al. [59]. All compounds demonstrated anticancer activities against HCT116, HT29, A549, H460, MCF-7, HeLa, HL60, and K562 cell lines at the low nanomolar level. Compound **15** (Figure 9), an *N*-phenyl derivative, was the most active among the pyrazolines and one of the most active overall especially against MCF-7 with a documented IC_50_ value of 0.21 nM. **15** was further evaluated in MCF-7 breast cancer mouse xenograft and showed in vivo efficacy at 10 mg/kg dose. All the synthesized derivatives displayed remarkably high affinity and selectivity towards cyclin-dependent kinase CDK1B compared to flavopiridol (Ki = 0.63 nM for **15**).

In a recent work, Miao et al., reported the discovery of simple *N*-phenyl pyrazoline-coumarin hybrids as novel heat shock protein (HSP90) inhibitors [60]. Compound **16** (Figure 9), the simplest unsubstituted *N*-phenyl and C-5 phenyl derivative, exhibited the best binding ability and inhibited the activity of HSP90 possessing at the same time an IC_50_ value of 4.7 μM for A549 lung cancer cell inhibition. This molecule proved to be more effective than their initially identified HSP90 inhibitor, a more complex coumarin-pyrazoline hybrid bearing a benzoic acid moiety [61]. The authors concluded that **16** not only induced apoptosis as their original inhibitor did, but also blocked autophagic flux in A549 cells.

Kumar et al. reported the synthesis of four *N*-phenyl pyrazoline-coumarin hybrids and their screening for anticancer activity against 60 cancer cell lines by NCI. Compound **17** (Figure 9) was found to be the most potent among the pyrazoline derivatives against non-small cell lung cancer NCI-H522. From the data obtained, it was observed that in this small series of four different substituents on C-5 phenyl ring, the simple non-hybrid coumarin compounds were more active than their hybrid analogues [62].

The *N*-unsubstituted (NH) pyrazoline-coumarin hybrid molecule **18** (Figure 9) was identified by Lesyk et al. [63] as the most potent cytotoxic compound in a series of 17 similar 6-pyrazolinylcoumarins showing sensitivity against CCRF-CEM and MOLT-4 cancer cell lines (GI_50_ values of 1.88 and 1.92 μM, respectively) after NCI 60-cell-line screening.

#### 5.2.2. Quinoline-Pyrazoline Hybrids

Quinolinones, nitrogen analogues of coumarins as well as the structurally similar quinolones and quinolinones comprise an important class of bioactive benzene-fused heterocycles. Quinolones are well-known for their antimicrobial activities as they have existed as clinical drugs for decades [64]. They are used as anticancer chemotherapeutics as well [65]. Some representative examples include, pelitinib, a 3-cyanoquinoline anticancer agent with irreversible EGFR inhibitory activity [66], neratinib, an EGFR inhibitor, used as an adjuvant therapy in patients with early stage breast cancer in which HER2 is overexpressed [67] and bosutinib, which is used for the treatment of chronic myelogenous leukemia [68].

**Figure 10:**
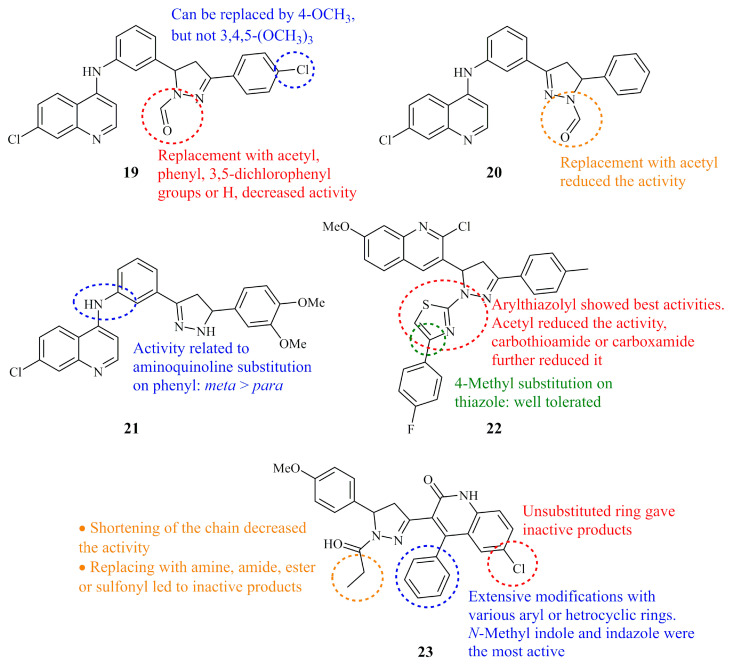
Chemical structures of anticancer quinoline and quinolinone-pyrazolines.

Similarly to the coumarins, quinolines are attached to the side C-3 or C-5 carbons of the 2-pyrazoline ring rather than to the N-1 nitrogen. Therefore, in the case of these hybrids, the N-1 nitrogen is available for further functionalization. Compound **19** (Figure 10), a hybrid molecule having formyl substituent on nitrogen at position-1 displayed high cytotoxic activity against a range of human cancer cell lines after screening by NCI [69]. Insuasty et al. reported GI_50_ values lower than 1.00 μM against a number of cell lines (0.39, 0.61, 0.40, 0.50, 0.38, 0.54, 0.64, 0.78, 0.98, 0.87, 0.82, and 0.28 μM for K562, MOLT-4, SR, SF-295, MDA-MB-435, SK-MEL-5, OVCAR-4, NCI/ADR-RES, A498, MCF-7, T-47D, and MDA-MB-468, respectively). No significant alteration of the GI_50_ values was observed for the analogue having a methoxy substituent at *para-* position of the C-3 phenyl ring instead of the chloride of **19**. *N*-Acetyl and *N*-phenyl pyrazoline analogues were screened as well, but demonstrated negligible activities. Earlier, the same group documented the synthesis and anticancer screening of similar *N*-acetyl and *N*-formyl derivatives with reverse substituents at C-3 and C-5 of the pyrazoline ring compared to **19** [70]. The formyl compounds were again by far the most active ones with GI_50_ values in many cancer cell lines under 1.00 μM (lowest GI_50_ value 0.13 μM against MDA-MB-435 for compound **20**) (Figure 10).

More recently, Charris et al. prepared and screened a number of closely related to **20** quinoline-pyrazoline hybrids with the main difference of the absence of a substituent at the nitrogen-1 of the pyrazoline ring [71]. Compound **21** (Figure 10) exhibited the best selectivity profile (human leukemia cells vs. human lymphocytes) with IC_50_ values of 3.17, 0.94, and 45.92 μM (24 h) against Jurkat E6.1, HL60, and normal lymphocytes.

A small library of thiazole bearing quinoline-pyrazoline conjugates were synthesized and evaluated as anticancer agents and EGFR inhibitors by George et al. [72], along with their pyrazoline and chalcone precursors. Compound **22** (Figure 10), a derivative consisting of all the three previously mentioned heterocyclic rings showed remarkable activity at nanomolar level (EGFR inhibition IC_50_ = 31.80 nM compared to the control gefitinib with IC_50_ = 29.16 nM). Regarding its cytotoxicity against cancerous cell lines, **22** displayed IC_50_ values of 0.227, 0.136, and 1.277 μM for MCF-7, HeLa, and DLD1, respectively. These values are significantly lower than those of both the pyrazoline precursor (1.155, 0.231, and 121.67 μM for the same cell lines) and the chalcone precursor (3.155, 2.100, 1.263 μM, respectively). However, it is worth mentioning that the EGFR inhibitory activity of the chalcone precursor was slightly worse than that of **22** (IC_50_ = 37.07 nM), whereas that of the pyrazoline precursor was not reported.

In a very recent report, Cavalli et al. identified a new class of RAD51-BRCA2 gene disruptors having quinolinone-pyrazoline hybrid structures [73]. After extensive structure–activity relationship studies they concluded that compound **23** was able to bind to its target (RAD51) and to inhibit the protein–protein interaction between RAD51 and BRCA2. According to their novel concept, **23** synergizes with olaparib to trigger synthetic lethality, the condition where simultaneous loss-of-function of the genes from complementary pathways result in loss of viability of cancer cells. However, they state that its low solubility may affect its metabolic and pharmacokinetic profile preventing it from advanced in vivo cancer model studies.

#### 5.2.3. Other Benzene-Fused Six Membered Heterocyclic Pyrazoline Hybrids

A limited number of hybrids with similar benzene-fused heterocycles have been reported. In all cases, the activities are preliminary or very low and no following investigations have been published.

**Figure 11:**
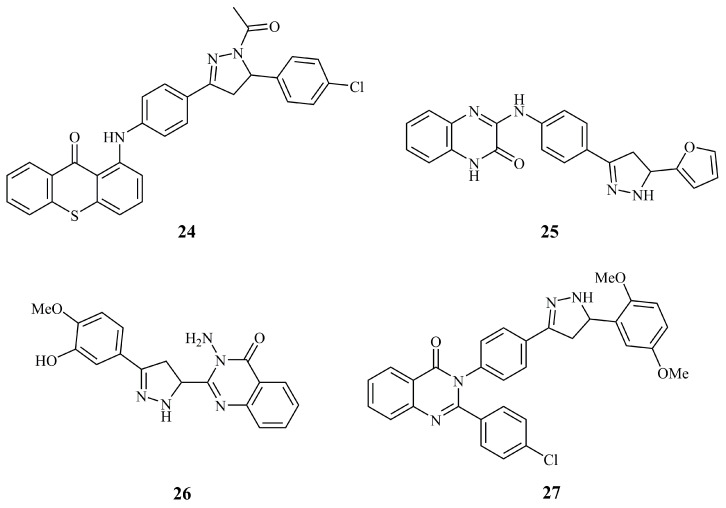
Chemical structures of anticancer pyrazoline hybrids with various benzene-fused heterocycles.

Thioxanthenone-pyrazoline conjugate **24** (Figure 11) was one of the thioxanthenone hybrids with various heterocycles and the most active when tested in vivo against L1210 leukemia in mice [74], but not much further information is provided. Other reported bioactivities for compounds **25**–**27** (Figure 11) are of less potential. These include the HepG_2_ cell growth inhibition of 76% at a concentration of 500 μg/mL of quinoxalinone **25** [75], and the activity against HepG_2_ cells by quinazolinones **26** exhibiting an IC_50_ value of 194 μM [76] and the 40.4% and 26.3% inhibition at 25 μM against MCF-7 and HepG_2_ for compound **27** [77].

### 5.3. Indole-Pyrazoline Hybrids and Other Benzene-Fused Five-Membered Heterocyclic Pyrazoline Hybrids

#### 5.3.1. Indole-Pyrazoline Hybrids

Indole belongs to a class of five-membered nitrogen heterocycles fused to a benzene ring. It is an important unit found in many natural and synthetic molecules with biological activity [78]. Indole nucleus is incorporated in several derivatives with anticancer activities, inhibiting at the same time the tubulin polymerization [79]. Vincristine, a complex molecule, prepared by semi-synthesis, is one of the earliest tubulin polymerization inhibitors [80].

**Figure 12:**
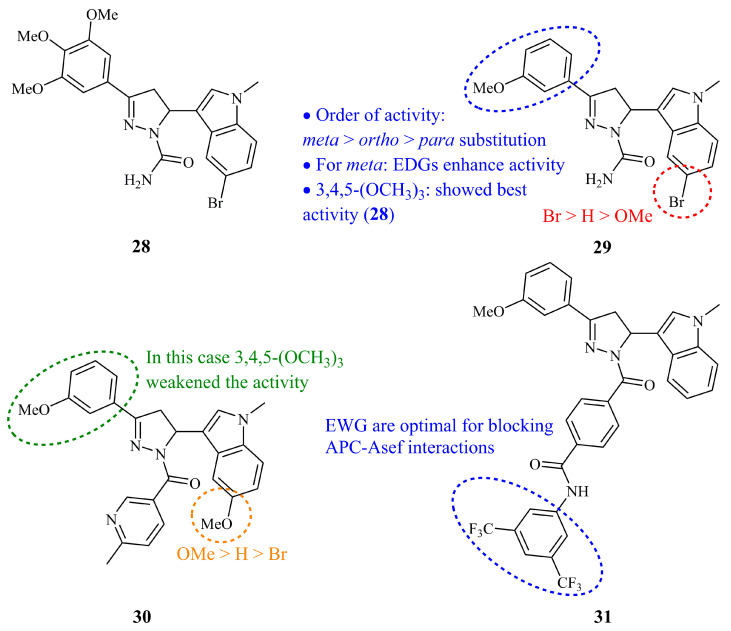
Chemical structures of anticancer indole pyrazoline conjugates [81].

In 2016, Zhu et al. published the first in a series of research articles on cytotoxic indole conjugated pyrazolines as potential inhibitors of tubulin polymerization [81]. The compounds were functionalized with a carboxamide group at N-1 of the pyrazoline ring. While most of the compounds showed significant activities, compound **28** (Figure 12) was the most active against both tubulin assembly (IC_50_ = 2.12 μM) and cancerous cell lines (IC_50_ values of 0.21, 0.29, 0.26, and 0.31 μM for HeLa, MCF-7, A549, and HepG2, respectively). Interestingly, it was the derivative bearing a 3-methoxy group at the phenyl substituent of the C-3 (compound **29,** Figure 12) and not the 3,4,5-trimethoxy one (compound **28**) that was selected from the authors for subsequent experiments. Actually, **29** displayed inhibitory activities at the same level with **28** (IC_50_ values of 2.44, 0.25, 0.32, 0.28, and 0.31 for tubulin polymerization and against HeLa, MCF-7, A549, and HepG_2_ cell lines, respectively). Compound **29**, hereafter named as YMR-65, was studied for its pharmacokinetic characteristics [82] and then for its pharmacodynamics in tumor-bearing mice. Briefly, the results demonstrated high tumor efficacy and low tissue damage [83].

Next, the same group expanded to *N*-nicotinoyl substituted analogues, keeping at the same time the main core of the previous structures almost unchanged [84]. According to the authors, this modification would improve the stability of the target molecules, making them less susceptible to oxidation. Compound **30** (Figure 12) exhibited most potency against cancer cell lines HeLa, HepG2, and MCF-7 with GI_50_ values of 0.034, 0.029, and 0.09 μM, respectively. It displayed high inhibitory activity against A549 (GI_50_ = 0.59 μM) as well, while it was found non-toxic to non-cancerous cells (CC_50_ > 300 μM). **30** was found to be more potent tubulin inhibitor (IC_50_ = 1.6 μM) than both **28** and the control combretastatin A4 (2.1 μM). The in vivo efficiency of **30** was evaluated on HeLa-xenograft mice and the results were comparable to combrestatin A4 (relative tumor ratio up to 61.52% without noticeable weight loss and tissue damage).

Following these findings, they further explored the abilities of this hybrid structure towards the blocking of adenomatous polyposis coli (APC)–Asef (the receptor of APC) interactions [85]. These interactions play a key role in proliferation and migration of human colorectal cancer [86]. The most active of these derivatives was (**31**) which exhibited high potency with IC_50_ value of 1.02 μM. Compound **31** is functionalized with an *N*-phenylbenzamide substituent (Figure 12). Finally, it was found that **31** showed selectivity against two out of the three human-derived colorectal cancer cell lines, the HCT119 and HT29, with GI_50_ values as low as 1.37 and 1.22 μM for, respectively, whereas it was much less active against other colorectal cells such as the CT26 for which GI_50_ value was 46.3 μM or other types of cells (19.7 μM for HeLa).

**Figure 13:**
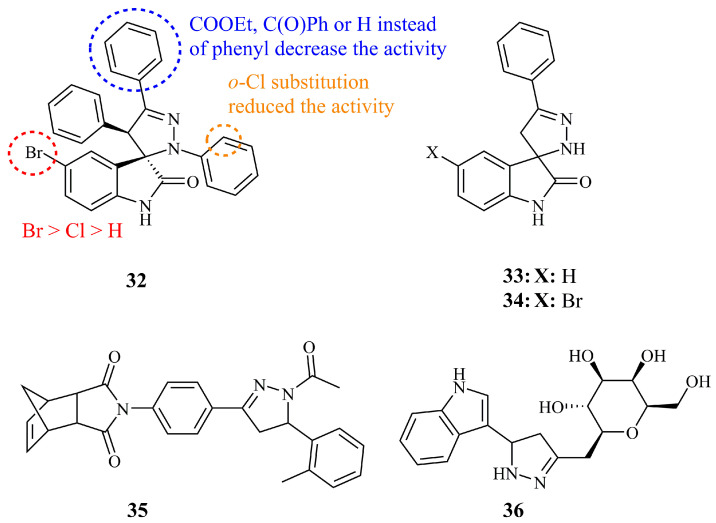
Chemical structures of anticancer oxindole and tetrahydro-methanoisondolodione-pyrazoline hybrids.

Santos and co-workers have published two research articles on the synthesis of novel spiropyrazoline oxindoles and their evaluation as anticancer agents against breast and colon cancer cells. Compound **32** (Figure 13) was identified as the most potent, being at the same time highly selective between MCF-7 and MDA-MB-231 tumor cells (GI_50_ values of 7.3 and >100 μM, respectively) [87]. **32** was found to be non-toxic against non-cancerous cell line HEK-293T (GI_50_ > 100 μM). The substituent of carbon-3 of the pyrazoline ring seemed to have a significant effect on the activity of the molecule. The presence of a substituted phenyl ring is crucial as these compounds are the only active (other include COOEt, COPh, and H). Moreover, the activity is dependent on the substituent of the aromatic ring of the oxindole following the order Br>Cl>H (GI_50_ values of 7.3, 37.7, and >100 μM for MCF-7, respectively). The authors mention that compounds with unsubstituted nitrogen at position 1 and carbon at position 4 of the pyrazoline ring are inactive, however, they do not report the activities of compounds having an unsubstituted N-1 and aryl substituted C-3 or vice versa. Next, they prepared a library of similar novel spiropyrazoline oxindole derivatives as inhibitors of human colon carcinoma cells [88]. Among the 23 products, compound **32** was once again one of the most active compounds against HCT-116 *p53* cell lines (IC_50_ = 13.1 μM) and was subjected to additional investigations. These newer derivatives exhibited more than 2-fold increased cytotoxicity compared to the initially developed spiroxazoline oxindole [89]. Additionally, the authors documented that combined therapy studies resulted in a synergistic effect of **32** with sub-toxic concentrations of the chemotherapeutic agent 5-FU on HCT-116 colon cancer proliferation. N-1 and C-4 spiropyrazoline oxindole derivatives were revisited by Gangarapu et al. in 2017 along with a series of thiadiazoline analogues [90]. While the latter proved to be more potent after screening by NCI, the unsubstituted **33** and the bromo analogue **34** (Figure 13) showed promising antitumor activity among the pyrazolines with GI_50_ (MG-MID) values of 6.02 and 4.07 μM, respectively. Moreover, in the same assay they exhibited GI_50_ values of 4.83 and 4.27 μM, respectively, against HCT-116 cell line, whereas they were slightly more cytotoxic against HOP-92 (GI_50_ values of 1.65 and 1.66 μM).

Novel *N*-acetyl pyrazolines bearing a tetrahydro-methanoisoindolodione unit have been synthesized and investigated for their anticancer activities by Kocyigit et al. Compound **35** (Figure 13) was found to be the most potent among 14 derivatives, displaying moderate IC_50_ value of 50.05 μM against C6 rat gliocarcinoma cells [91].

Pyrazoline-bridged indole C-glycoside **36** (Figure 13) displayed low micromolar and selective cytotoxicity against MCF-7 (IC_5_0 = 4.67 μM) compared to other breast cancer cell lines [92]. As a matter of fact, **36** was less active against MDA-MB-231 (IC_50_ = 35.5 μM) and far less active against MDA-MB-453 (IC_50_ not determined—cell viability was determined at 69.6% at 25 μM). Compound **36** demonstrated very low cytotoxicity against healthy breast cell line MCF-10A (cell viability for maximum concentration tested: 82.4% at 100 μM). The authors reported inhibition of COX-2 enzyme as well, which was claimed to be at least partially responsible for the cytotoxicity observed. Interestingly, isoxazole analogues showed better cytotoxity against MCF-7 with IC_50_ values lower than 1 μM.

We will see in the Section 5.4.2, the development of three-heterocycles-containing indole-pyrazolines with compound **53** being the most potent after screening against 60 cancerous cell lines by NCI. For classification reasons, **53** is classified as thiazolidinone hybrid since that heterocyclic ring is the one attached directly to the pyrazoline.

#### 5.3.2. Isatin-Pyrazoline Hybrids

**Figure 14:**
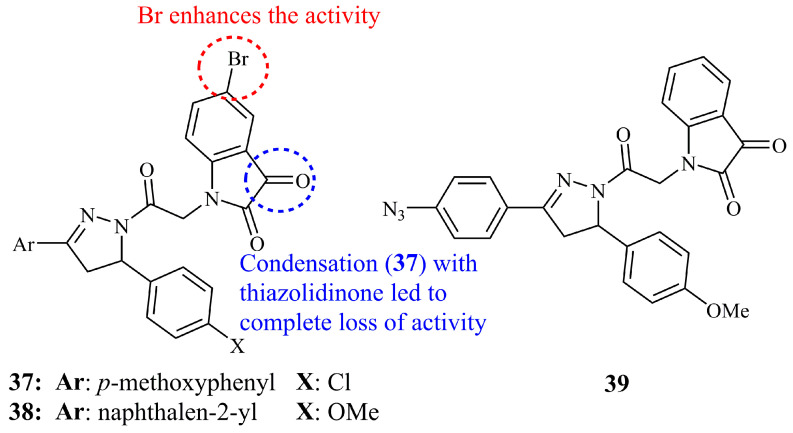
Chemical structures of anticancer isatin-pyrazoline hybrids.

Isatins (1*H*-indole-2,3-diones), also known as indenediones and indole quinones are indole derivatives having two carbonyl groups at positions 2 and 3 [93]. Compound **37**, a hybrid molecule containing an isatin unit attached to the pyrazoline via a 2-oxoethyl linker, was found to be the most active against leukemia cancer cell lines (GI_50_ values of 0.69–3.35 μM) among a series of products synthesized and evaluated by Havrylyuk and Lesyk [94]. Characteristic substituents of **37** (Figure 14), include the *para*-methoxyphenyl ring at C-3 of the pyrazolinic ring and the familiar from some active indole based pyrazolines (see Section 5.3.1) bromine atom at C-5 of the isatine subunit. In this work, a number of analogues containing an additional thiazolidinone ring were demonstrated, but their activities were mostly insignificant. Following these findings, compound **38** (Figure 14) was reported by the same authors, displaying high activity against leukemia cancer cell lines (GI_50_ values of 5.49–5.75 μM), nevertheless it was less active than the former set of compounds [95].

The phenylazide isatine-pyrazoline hybrid **39** (Figure 14) was found active against NCI-H23 and MCF-7 (IC_50_ values of 1.06 and 3.92 μM, respectively) among five analogues. The in vivo tests showed good correlation with the in vitro screening, revealing that **39** was the most effective on survival time (31.5 days) on Ehrlich ascites carcinoma (EAC) bearing mice with the highest increase in life span (ILS = 73.47%), accompanied with maximum reduction of viable cells and tumor volume [96].

#### 5.3.3. Other Benzene-Fused Five-Membered Heterocyclic Pyrazoline Hybrids

**Figure 15:**
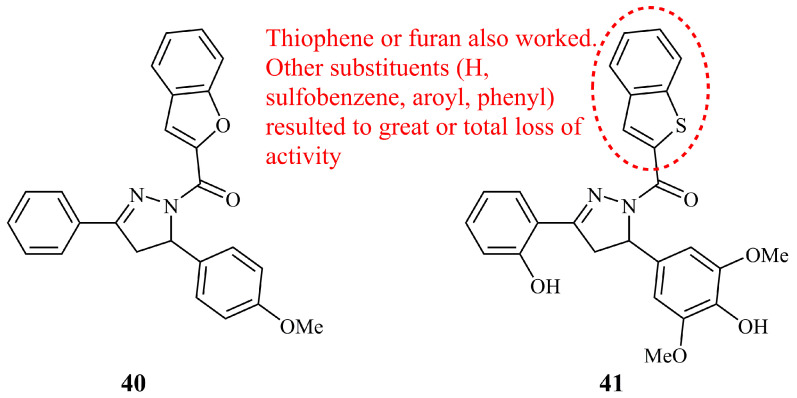
Chemical structures of anticancer oxygen and sulfur analogues of indole-pyrazoline hybrids.

Benzofuran and benzothiophene are the oxygen and sulfur analogues of indole. Similarly to indole, they are comprised of a benzene ring fused to a furan or thiophene ring. Compound **40** (Figure 15) was found to be the most potent against A2780*cis* cell line (IC_50_ = 4.569 μg/mL) while being the second most active as reversal agent of human MDR1-gene transfected mouse lymphoma cell line among 24 novel analogues [97]. Benzothiophene-pyrazoline hybrid **41** (Figure 15) was the most effective anticancer agent in a series of 19 novel compounds with different substituents at the nitrogen-1 of the pyrazoline (sulfobenzene, aroyl, phenyl, and others) [98]. It demonstrated low IC_50_ value of 3.57 μM after 24 h exposure against Hep-G_2_ (lower than control cisplatin with IC_50_ = 8.45 μM) and low cytotoxicity against primary hepatocytes (IC_50_ = 33.47 μM). HepG_2_ cells treated with **41** could be arrested in the G_2_/M phase. The analogues of **41** bearing a thiophene or furan ring instead of benzothiophene were closely active (IC_50_ values of 4.51 and 4.61 μM against HepG_2_, respectively, and 19.24 and 20.73 μM against primary hepatocytes).

Meanwhile tetrahydrocarbazole based pyrazolines, unsubstituted at N-1 of the pyrazoline ring displayed none or insignificant anticancer activities [99].

### 5.4. Thiazole and Thiazolidinone Pyrazoline Hybrids

#### 5.4.1. Thiazole and Benzothiazole Pyrazoline Hybrids

Thiazoles are versatile compounds found in numerous natural products such as the microbial secondary metabolites epothilones A and B [100] and the cyclic peptides urukthapelstatin A [101], aerucyclamides A–D [102], and hexamollamide [103], as well as in commercial drugs, for example the antiretroviral ritonavir [104], the antifungal abafungin [105], and the antibiotic sulfathiazole [106]. Since thiazoles exhibit noteworthy cytotoxic activities [107,108], the development of their pyrazoline conjugates [109] seems a reasonable and promising strategy for potential anticancer agents.

**Figure 16:**
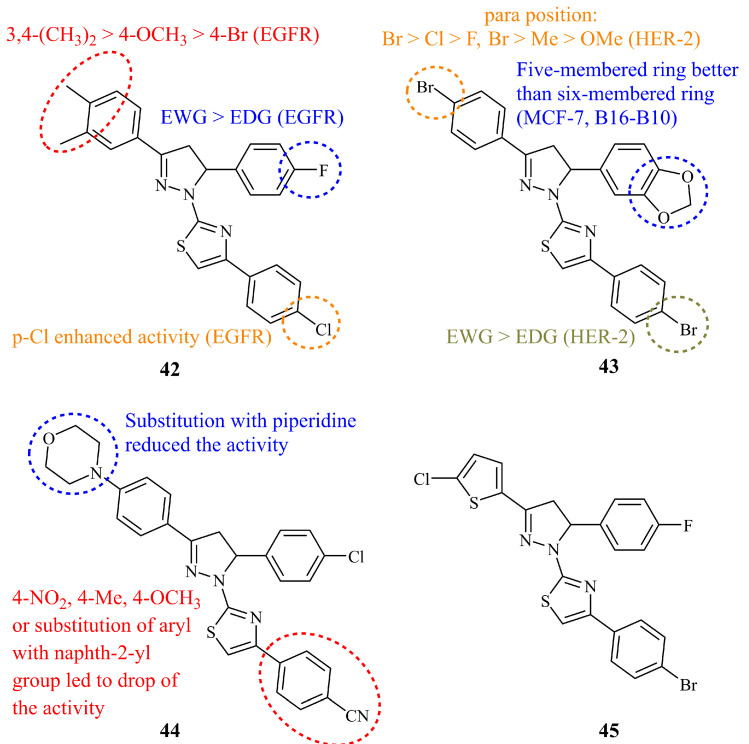
Chemical structures of anticancer thiazolyl-pyrazolines.

The first approach was made by Zhu et al. in 2011 when 42 thiazolyl-pyrazoline compounds were screened for their antiproliferative and EGFR kinase inhibitory activity [110]. Some of the derivatives were, indeed, potent inhibitors of EGFR kinase, with compound **42** (Figure 16) displaying both the highest activity against both EGFR and MCF-7 cytotoxicity (IC_50_ values of 0.06 and 0.07 μM, respectively). Structure-activity relationships revealed that the presence of the two methyl groups at positions 3 and 4 of the C-3 phenyl ring, increased remarkably the EGFR inhibitory activity. In their follow up work, they prepared a series of analogues containing a benzodioxole at C-5 instead of a substituted aryl group. Compound **43** (Figure 16) showed high antiproliferative activity with IC_50_ values of 0.09 and 0.12 μM against MCF-7 and B16-F10 cell lines, respectively [111]. In this work, the authors chose HER-2 kinase to screen the products against and perform docking simulations as it is the homologous protein of human EGFR. Again, compound **43**, was the most active (IC_50_ = 0.18 μM).

Recently, Altintop et al. reported the synthesis and evaluation of a library of 26 thiazolyl-pyrazoline hybrids as dual EGFR and HER2 inhibitors, incorporating the morpholine or piperidine ring as well [112]. The morpholine bearing derivative **44** (Figure 16) showed both the highest EGFR and HER2 inhibitory activity with IC_50_ values of 4.34 and 2.28 μM. However, the observed IC_50_ values were still two orders of magnitude less active than clinically used positive control erlotinib (IC_50_ = 0.05 μM for EGFR). Compound **44** was also the most active against A549 and MCF-7 cell lines (IC_50_ = 10.76 and 8.05 Μm, respectively). In earlier study by the same group, compound **45** (Figure 16) was found to be the most active against A549 in a series of 22 hybrids (IC_50_ = 62.5 μg/mL) displaying at the same time low cytotoxicity against NIH/3T3 mouse embryonic fibroblast cell line [113].

**Figure 17:**
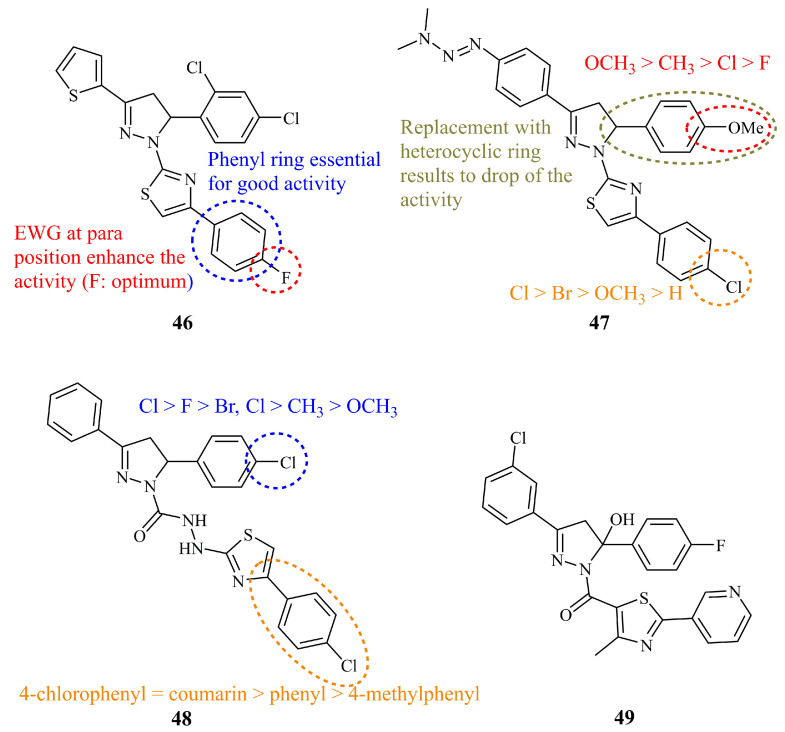
Chemical structures of anticancer thiazolyl-pyrazolines.

Edrees et al. documented recently the synthesis and anticancer screening against HepG_2_ human hepatocellular carcinoma cell line of a library consisting of 19 pyrazoline derivatives bearing a thiophenyl group at C-3 [114]. The structures were divided in three groups depending on the exact formula of the thiazole unit; thiazoles incorporating an acyclic nitrogen-nitrogen double bond group, thiazolones, and arylsubstituted thiazoles. The latter showed the most potent activity by far (IC_50_ = 1.70 μM for compound **46**) (Figure 17). The activity was dependent on the substituent at the 4-position of the phenyl ring as fluoride was the most effective, followed by chloride (IC_50_ = 2.98 μM) whereas when these atoms were replaced by the electron donating methyl group, the IC_50_ value increased to 14.91 μM.

Similar compounds with a thiazole ring attached to N-1 of the pyrazoline nucleus were synthesized by Ravula et al. These compounds are differentiated from the previous ones predominately by a dimethyl triazene group bonded to the phenyl ring at C-5 [115]. Structure-activity relationships revealed that the chloride group combined with the methoxy group at the 4-positions of the phenyl groups (compound **47**) (Figure 17) increased the activity against MCF-7 and HT-29 (IC_50_ values of 9.04 and 10.65 μg/mL, respectively). The replacement of the phenyl group attached to C-3 of the pyrazoline ring by a thiophene group decreased the activity substantially.

A slightly different approach to thiazole hybrids was followed by Sadashiva et al. and by Santosh et al. in two recently published articles. The first group synthesized a series of thiazoles linked through a (hydrazino)carbothioamide linker to the pyrazoline [116]. Among them, compound **48** (Figure 17) showed similar to or better activity than the positive control cisplatin against A549 and MCF-7 cancer cell lines (IC_50_ 5.0 (10.0) and 7.5 (7.5) μM, respectively—in parentheses is the IC_50_ values of cisplatin). The chloride atom at the 4-position of the C-5 phenyl ring proved beneficial for the activity versus bromide, methoxy, methyl, and fluoride. No significant alterations in activities were observed for a coumarin substituent at the thiazole ring instead of the 4-chlorophenyl. On the other hand, Santosh et al. prepared a series of 12 hydroxyl pyrazolines bearing a thiazole unit attached via a carbonyl group to the N-1 of the pyrazoline [117]. All compounds showed low to moderate activities. Compound **49** (Figure 17) displayed the lowest IC_50_ values against MDA-MB-231 (24.78 μM) and HT-29 (26.64 μM).

**Figure 18:**
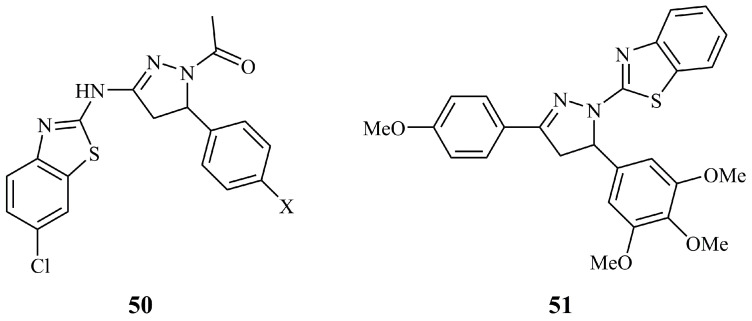
Chemical structures of benzothiazolyl-pyrazolines tested for their anticancer activity.

Two *N*-acetyl pyrazoline derivatives (**50**, X = -OMe or -Cl) of aminobenzothiazole connected at the C-3 of the pyrazoline ring (Figure 18), screened by NCI among other aminobenzothiazole compounds were until recently the only structures containing a fused thiazole [118]. However none of them showed any remarkable activity; the most active was their α,β-unsaturated ketone precursor. Very recently, the novel compound **51** (Figure 18), having a benzothiazole moiety linked directly to the N-1 of the pyrazoline ring, was found to exhibit moderate activity against human squamous carcinoma cell lines Ca9-22, HSC-2, HSC-3, and HSC-4 with CC_50_ values of 10.8, 26.0, 16.5, and 21.4 μM, respectively. At the same time, it was found non-toxic against human normal oral cells with CC_50_ values ranging from 301.7 to 315.7 μM [119].

#### 5.4.2. Thiazolidinone-Pyrazoline Hybrids

Thiazolidinone is a biologically important five-membered heterocyclic ring having two heteroatoms; a sulfur at position 1 and a nitrogen at position 3 along with a carbonyl group at positions 2, 4, or 5 [120]. Besides the relative thiazolidine-2,4-diones which bear two carbonyls on their ring, the five different forms of thiazolidinones exhibit a wide range of biological applications as antitubercular [121], antimicrobial [122], antiviral [123], and anticancer agents [124], making them an attractive structure for molecular hybridization.

**Figure 19:**
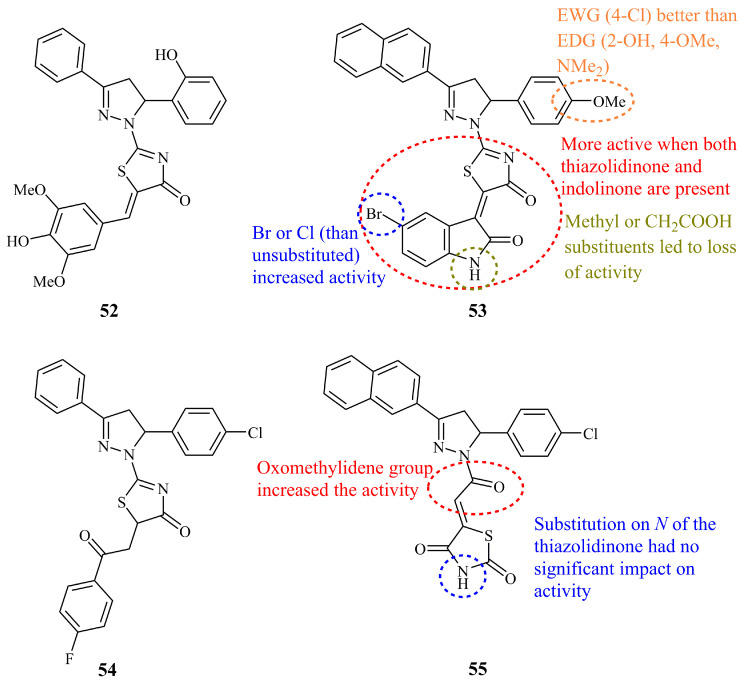
Chemical structures of anticancer thiazolidinone-pyrazolines.

Probably the most influencing studies on thiazolidinone bearing pyrazoline hybrids with interesting cytotoxic activity have been published by Havrylyuk and Lesyk. In 2009, they reported the synthesis of a library of mostly arylidene and a few unsubstituted thiazolone conjugates [125]. The in vitro anticancer activity was tested by NCI and compound **52** (Figure 19) was found to be the most active against colon cancer cell line HT-29 and non-small cell lung cancer cell line HOP-62 with logGI_50_ values of −6.37 and −6.12. After rational design and optimization, they prepared compound **53** (Figure 19), which was screened along with 39 similar compounds and displayed mean GI_50_ and TGI values of 0.071 and 0.76 μM, respectively, which reflect a substantial improvement of activity compared to compound **52** (GI_50_ and TGI values of 2.45 and 7.24 μM) [126]. More specifically, **53** showed high activity against HOP-92 (GI_50_ < 0.01 μM), HCT-116 (GI_50_ = 0.018 μM), SNB-75 (GI_50_ = 0.0159 μM), NCI/ADR-RES (GI_50_ = 0.0169 μM), and RXF 393 (GI_50_ = 0.0197 μM). In structural terms, compound **53** is a non-condensed hybrid molecule, containing thiazolidinone, pyrazoline, and indolone moieties. Once again, the presence of a methoxy group at position 4 of the C-5 phenyl ring proved crucial for the activity. Analogues containing pyrazole unit instead of the pyrazoline, without the presence of a thiazolone ring, demonstrated significant decrease of the activity. Next, they reported the synthesis of hybrids containing aryl-oxo-ethyl substituted thiazolinone, their corresponding amides, and some simpler hybrid structures, concluding to the most active among them, compound **54** (Figure 19), which selectively inhibits the growth of leukemia cell line RPMI (GP = 63.60% at 10^−5^ M concentration) [127]. As an extension of their previous works, the same authors prepared and screened a series of thiazolidine-2,4-dione analogues [128]. Compound **55** (Figure 19) showed the highest activity against leukemia subpanel cell lines with GI_50_ values of 1.64–3.20 μM.

**Figure 20:**
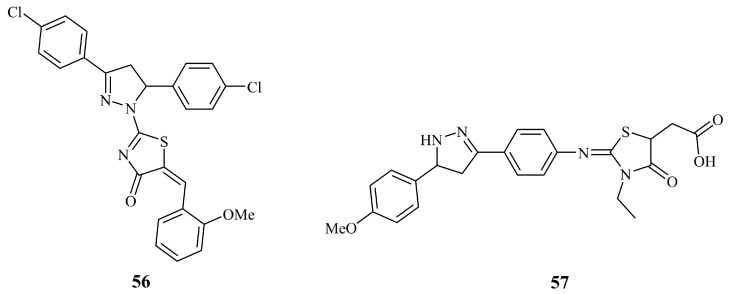
Chemical structures of anticancer thiazolidinone pyrazoline conjugates.

Compound **56** (Figure 20) was found to be the most potent against MCF-7 cancer cell lines (IC_50_ = 1.4 μM) among 12 5-arylidenethiazolone bearing pyrazolines [129]. This value was less than half of the value obtained for the positive control doxorubicin (IC_50_ = 2.9 μM). Compound **57** (Figure 20), an *N*-unsubstituted pyrazoline that is conjugated with a thiazolone moiety via a phenyliminic moiety at C-3 of the pyrazoline ring, displayed moderate (IC_50_ = 3.22 μM) activity against the MCF-7 cell line. Doxorubicin was used as control, and its reported IC_50_ was at the range of 0.45 μM [130].

### 5.5. Two Nitrogen Containing Heterocyclic–Pyrazoline Hybrids and Analogues

#### 5.5.1. Pyrazole-Pyrazoline Hybrids

Pyrazoles are the aromatic analogues of pyrazolines. They have been investigated extensively for their biological activities [131]. Many methods have been developed for the synthesis of functionalized pyrazoles giving medicinal chemists the opportunity to approach a wide variety of structures [132]. In many cases, they can be directly furnished from the corresponding pyrazolines via oxidative aromatization [133].

**Figure 21:**
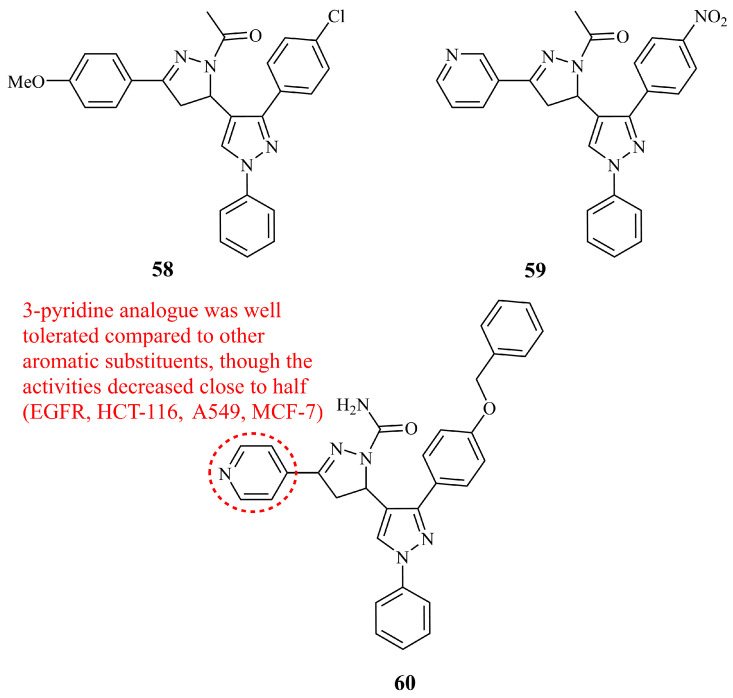
Chemical structures of anticancer pyrazole-pyrazoline hybrids.

Insuasty et al. prepared two series of *N*-acetyl and *N*-unsubstituted pyrazolines attached to a functionalized pyrazole directly from carbon-5 of the pyrazoline ring [134]. Selected compounds from these libraries, along with some of their pyrazolic chalcone precursors were screened by NCI for their inhibitory activity against 60 human tumor cell lines. Compound **58** (Figure 21) was the only hybrid molecule denoted as active. It exhibited moderate to high activity for non-small cell lung cancer (GI_50_ values 0.04–2.20 μM), leukemia (GI_50_ values 1.57–1.73 μM), renal (GI_50_ values 1.82–1.96 μM), and breast (GI_50_ values 1.88–1.90 μM). GI_50_ values for MDA-MB-231, HCT-116, SR, and HOP-62 were calculated at 1.88, 1.94, 1.57, and 0.04 μM, respectively. Interestingly, similar activity was obtained from one of the pyrazolic chalcone precursors, though not the one with the same substituents as **58**.

Two series of pyrazolyl pyrazolines and pyrazolyl aminopyridines were synthesized and evaluated for their *in vitro* cytoxicity against the human cancer cell lines HeLa, NCI-H460, and PC-3 [135]. The aminopyridine derivatives proved to be at least two times more active than the pyrazolines. Among the latter, compound **59** (Figure 21) was found the most active with relatively low cytotoxicity against non-cancerous cell lines (IC_50_ values of 11.46, 13.41, 22.47, and 82.45 μM for HeLa, NCI-H460, PC-3, and the non-cancerous NIH-3T3, respectively).

While keeping the core pyrazolyl pyrazoline structure unchanged, Nawaz et al. introduced a bulky benzyloxy group in the place of NO_2_ and Cl of the previous most active structures and a carboxamide group instead of an acetyl at nitrogen-1 of the pyrazoline aiming to develop EGFR kinase inhibitors [136]. Compound **60** (Figure 21) was the most active (IC_50_ 3.65 μM), being at the same time the most potent against four different human cancer cell lines (IC_50_ values of 6.5, 4.6, 12.6, and 40.8 μM against HCT-116, A549, MCF-7, and SiHa, respectively). Interestingly, although 16 different substituents at C-3 of the pyrazoline ring were introduced, the most effective was the 4-pyridine (compound **60**), followed by 3-pyridine, giving a good correlation with the compound **59**.

#### 5.5.2. Imidazole and Benzimidazole Pyrazoline Hybrids

The proved anticancer potential of both imidazoles and benzimidazoles led to the design of pyrazoline/(benz)imidazole conjugates as anticancer agents.

**Figure 22:**
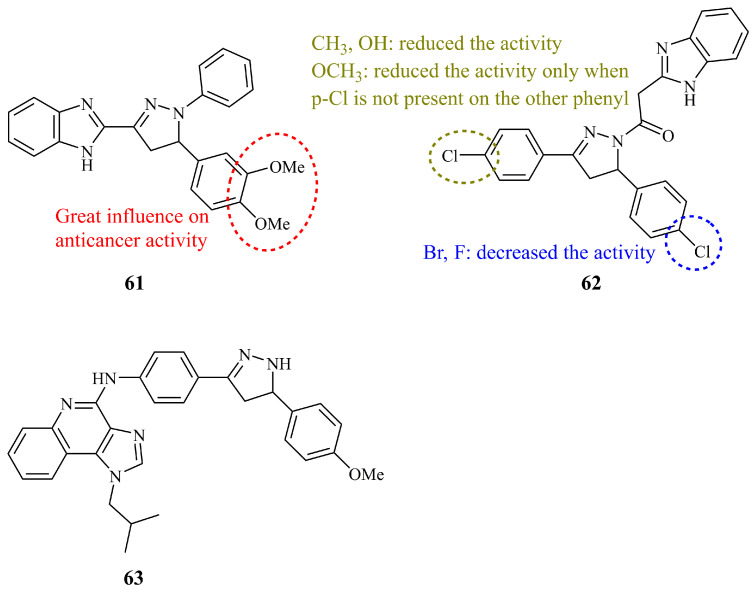
Chemical structures of anticancer imidazole containing pyrazoline hybrids.

In the first attempt, among the 14 synthesized benzimidazolyl pyrazolines, six were selected by NCI for screening against a panel of 60 cancer cell lines [137]. Benzimidazole unit was linked directly to the carbon-3 of the pyrazoline ring. After obtaining the initial screening results of both *N*-unsubstituted and *N*-phenyl pyrazoline hybrid derivatives, compound **61** (Figure 22) was found to be the most active and meeting the criteria for further evaluation at five dose level screening. The authors concluded that the role of the methoxy substituents on the phenyl group at position-5 of the pyrazoline was crucial for antiproliferative activity. Whereas, overall, **61** was found to be non-selective, it exhibited moderate to high inhibitory activity against most breast cancer cells (representative GI_50_ values: 2.41, 7.19, and 3.09 μM for MCF-7, MDA-MB-231, and T-47D, respectively).

Compound **62** (Figure 22), having a benzimidazole moiety connected via an oxoethyl linker to the N-1 of the pyrazoline, showed effective growth inhibition against the lung cancer cell line A549 (IC_50_ = 2.2 μM) and EGFR binding affinity (IC_50_ = 0.97 μM) [138]. It was found that **62** inhibited growth of A549, by inducing a strong G_2_/M phase arrest. In addition, the analogue having a methoxy substituent at *para* position of the C-3 phenyl ring displayed activity comparable to that of **62** (IC_50_ values of 2.8 and 1.7 μM for A549 and EGFR, respectively).

Apart from these efforts, a very limited number of related compounds have been prepared and reported which, however, have either shown no activity at all, being far less active than their precursors [139], or they display IC_50_ values over 40 μM against tested cell lines [140], or, in some cases, their activities are not reported at all, presumably due to the lack of it [141]. Nevertheless, compound **63** (Figure 22) incorporating a structurally interesting imidazoquinonyl moiety has shown low cytotoxicity against HeLa cancer cells (IC_50_ = 15.1 μM), slightly higher than its chalcone precursor or *N*-phenyl pyrazoline analogue [142]. The activity decreased significantly when the authors replaced the methoxy group of the C-5 phenyl ring with a nitro or chloride substituent or when they left the ring unsubstituted.

#### 5.5.3. Other Two Heteroatom Heterocyclic Pyrazoline Hybrids

**Figure 23:**
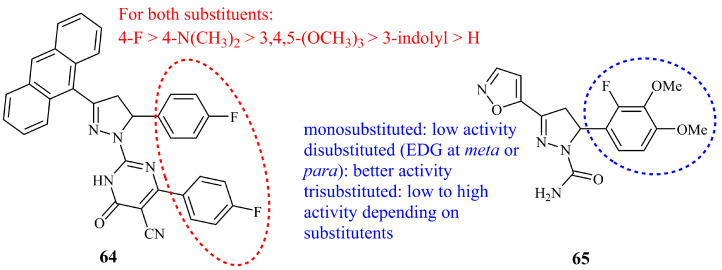
Chemical structures of anticancer pyrimidine and oxazoline containing pyrazoline hybrids.

Recently, pyrimidinone-pyrazoline-anthracene derivatives were synthesized and evaluated as potential anticancer agents for hepatocellular carcinoma [143]. Among 20 derivatives and their pyrimidinone and chalcone precursors, compound **64** (Figure 23) displayed the best activities overall, particularly in terms of cancerous vs. normal cells growth inhibition. **64** was found more cytotoxic against HepG_2_ with IC_50_ value of 4.22 μg/mL equal to 7.2 μM (IC_50_ = 33.14 μg/mL against fibroblast cells) whereas it was less active against Huh-7 cell line (IC_50_ = 8.45 μg/mL). These values may represent low to moderate activities, but they are significantly better than those of compounds **26** and **27** (see Section 5.2.3) bearing a similar benzene-fused ring. Analogues of **64** having a *p*-dimethylamino or 3,4,5-trimethoxy substituents instead of *p*-fluoro on C-5 phenyl ring displayed better activities against Huh-7 cell line (IC_50_ = 6.13 and 4.78 μg/mL, respectively). In any case, these values are comparable to those of control doxorubicin (IC_50_ = 5.43 and 6.40 for HepG_2_ and Huh-7, respectively).

It was previously mentioned (see Section 5.3.1) that oxazolines exhibited inferior anticancer activities compared to their pyrazoline analogues as exemplified by **32** in the case of oxindole hybrids. However, chalcone derived isoxazole-pyrazoline hybrids have displayed interesting cytotoxic activities [144]. Compound **65** (Figure 23) was found to be the most potent among 15 analogues against human prostate cancer cell DU-145 (IC_50_ = 2 μg/mL) while it was non-toxic (IC_50_ > 40 μg/mL) against normal human cells (L02). This activity was 2.5 times better than the isoxazole chalcone precursor (IC_50_ = 5 μg/mL, while still non-toxic against L02). From the structural point of view, compound **65** has the isoxazole moiety directly attached to C-3 of the pyrazoline ring and the N-1 is substituted with a carboxamide group.

### 5.6. Three or More Nitrogen Heterocyclic–Pyrazoline Hybrids and Analogues

#### 5.6.1. Triazole-Pyrazoline Hybrids

Fluconazole, a very potent and widely used bis-(1,2,4)-triazole antifungal drug [145], which may represent the most easily recognizable active triazole to chemists, it is indeed only one of the variety of examples of all three triazole isomers possessing potent and diverse biological activities. The derivatives that have been used for hybridization with pyrazoline framework are so far only 1,2,3-triazoles. Presumably by choice, since many 1,2,3-triazole-based hybrids have been presented as lead compounds for various applications [146], and 1,2,3-triazole containing compounds, such as cefatrizine and carboxyamidotriazole, have already been used in clinic or are under evaluation for cancer treatment [147].

**Figure 24:**
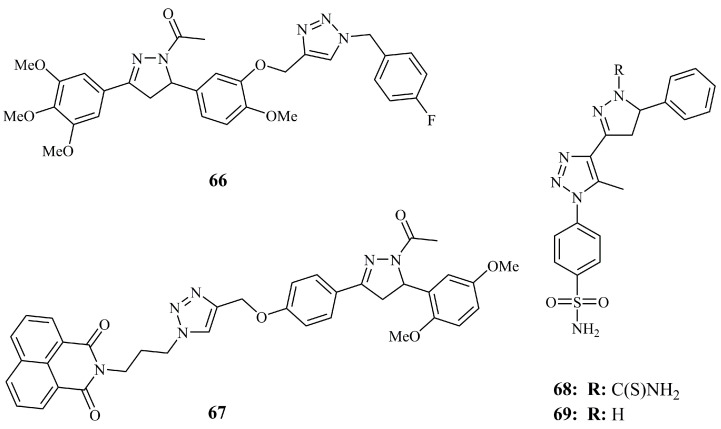
Chemical structures of anticancer triazole containing pyrazoline hybrids.

Compound **66** (Figure 24) inhibited HepG_2_ cell line most effectively (GI_50_ = 6.7 μM) and displayed consistent inhibitory effect on the other three cell lines tested (GI_50_ < 10 μM for HeLa, DU145 and A549) among 16 analogues with combretastatin-A4 alike substituents conjugated with triazole [148]. Moreover, it caused accumulation of cells in G_2_/M phase and inhibited tubulin polymerization (36% inhibition at 3 μM). Interestingly, the chalcone precursors with the same substituents displayed significantly better inhibitory activities.

Recently, in order to develop potential selective estrogen receptor modulators (SERMs), Kumar et al., synthesized a number of triazole tethered naphthalimide pyrazoline conjugates [149]. Along with docking simulations, they evaluated the cytotoxic activity of pyrazolines and their chalcone precursors against MCF-7 and MDA-MB-231 using MTT assay and plumbagin as the positive control. Only compound **67** (Figure 24) showed inhibitory activity (expressed as IC_50_) well under 100 μM (62.23 μM), which is not high, but still comparable to that of the standard drug tamoxifen (IC_50_ = 50 μM). **66** was inactive against both MDA-MB-231 and non-cancerous HEK-293 cells (IC_50_ values over 100 μM).

*N*-Carbothioamide pyrazoline **68** (Figure 24), incorporating a triazole benzene sulfonamide group at C-3 of the pyrazoline ring was found to be the most cytotoxic overall against HeLa and MCF-7 (IC_50_ values of 8.33 and 11.08 μg/mL, respectively) among two series of carbothioamide pyrazoline and isoxazoline analogues [150]. As a matter of fact, the analogue having a chloride substituent at *para* position of the C-5 phenyl ring exhibited lower activity for MCF-7 (IC_50_ = 8.07 μg/mL). Almost simultaneously, another group reported the poor anticancer activity against MCF-7 (IC_50_ = 99.54 μM) for compound **69** (Figure 24) which differs to **68** only by the carbothioamide group [151].

#### 5.6.2. Triazine-Pyrazoline Hybrids

Out of the three possible triazine isomers, only the 1,3,5-triazine and the 1,2,4-triazine rings have been incorporated in a pyrazoline hybrid structure so far. The former, also referred to as *s*-triazine (from symmetric), has been only reported in three studies. Derivatives of this six-membered heteroaromatic structure, both uncondensed and hetero-fused, have shown interesting antitumor activities and some of them, have reached clinical development, like the (+) enantiomer of 4-(4-fluoro-2-methoxyphenyl)-N-{3-[(S-methylsulfonilimidoyl)methyl] phenyl}-1,3,5-triazin-2-amine, which is currently in clinical development for the treatment of Acute Myeloid Leukemia (AML) [152]. However, it is the 1,2,4-isomer that has been studied most extensively, as many derivatives bearing this ring have exhibited remarkable antitumor activities and have been patented and reached advance phases of clinical trials. Tirapazamine is an example of such derivatives, also known as SR-4233, an experimental drug that is activated in hypoxic conditions and used in hypoxic regions of head, neck, and gynecological solid tumor cancer types, resistant to radiotherapy and most anticancer drugs [153].

**Figure 25:**
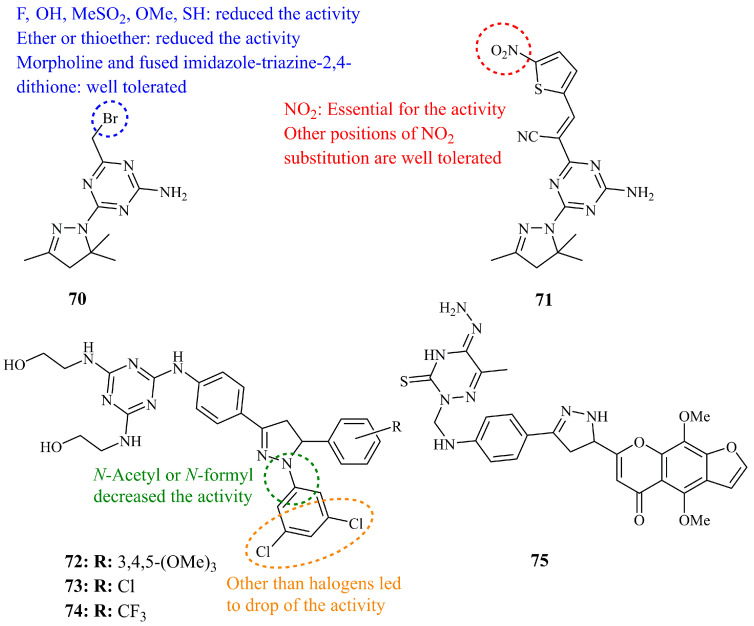
Chemical structures of anticancer triazole containing pyrazoline hybrids.

A series of 2,4-diamino-1,3,5-triazine derivatives were synthesized by Brzozowski et al. and screened by NCI for their inhibition activity against a panel of cancer cell lines. Compound **70** (Figure 25), a compact and low molecular weight pyrazoline hybrid showed the most potent activity with mean midpoint values of log_10_ GI_50_, log_10_ TGI_50_, and log_10_ LC_50_ equal to −5.26, −4.81, and −4.37, respectively [154]. Subsequent studies and evaluation by the same group led to the identification of compound **71** (Figure 25) having a more “sophisticated” cyanovinyl substituent at position 6 of the triazine ring, in the place of bromomethyl group [155]. Mean midpoint value of log_10_ GI_50_ was calculated at −6.59.

More recently, Insuasty et al. reported the synthesis and evaluation of a series of chalcone derived 1,3,5-triazine containing 2-pyrazolines. Seventeen of them were selected and screened by NCI, where compounds **72**–**74** (Figure 25) were proved to be the most potent of the library, along with the chalcone precursor of **74** [156]. The lowest GI_50_ value was determined for **72** against renal cell line RXF-393 (0.569 μM) and breast HS-578 (0.644 μM). For comparison purposes, since we have mentioned the activities of many compounds against MCF-7, MDA-MB-231, and HCT-116 the corresponding values found were 1.26, 1.45, and 1.28 μM, respectively, for compound **72**, 1.42, 1.71, and 1.59 μM, respectively, for compound **73** and, finally, 1.27, 1.63, and 1.55 μM for compound **74**.

A series of ten 3-thioxo-1,2,4-triazines bearing pyrazolines among other heterocycles (oxazoles and pyridines), was prepared and evaluated against various cancer cell lines in vitro and in vivo [157]. The authors claimed that the IC_50_ values of most compounds, and, in particular **75** (Figure 25) which found as one of the most active ones, against cancer cell lines like KB, SKOV-3, HL-60, U937, G361, HeLa, MCF-7, and others, were determined at picomolar levels. These results prompted the research group to perform *in vivo* experiments against MCF-7 mouse xenograft models of breast cancer. Compound **75** showed marked acute activity, whereas the starting material 3,4-dihydro-6-methyl-3-thioxo-1,2,4-triazin-5(2H)-one, used as control, was the least active. As an example, the ratio Vt/Vo for the tumor growth was found to be 1.15, 1.75, 2.37, and 4.31 after 2, 8, 14, 20 days, respectively, for compound **75** and 1.38, 9.88, 27.66, and 40.21 after 2, 8, 14, 20 days, respectively, for the starting triazine.

#### 5.6.3. Additional Three or More Heteroatom Heterocyclic–Pyrazoline Hybrids and Analogues

**Figure 26:**
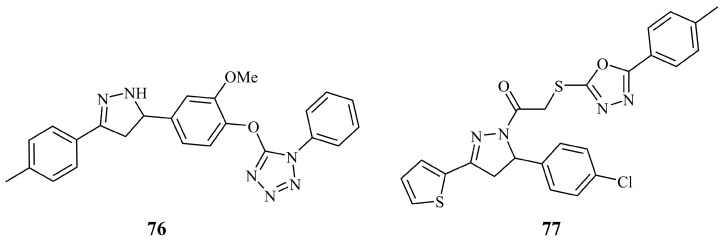
Chemical structures of anticancer a tetrazole and an oxodiazole containing pyrazoline hybrids.

Increasing the number of nitrogens in the second heterocyclic component of pyrazoline hybrids further led to the development of tetrazole-pyrazolines by El-Monaem et al. [158]. Most of the target hybrid structures were inactive or at least less active than their chalcone precursors. Only pyrazoline **76** (Figure 26) displayed encouraging activities against HCT-116, PC-3, and MCF-7 (IC_50_ values: 8.0, 16.5, and 15.6 μg/mL, respectively) while being inactive against normal green monkey kidney cell line Vero B (IC_50_ > 50 μg/mL). Özdemir et al. synthesized a series of 12 pyrazoline hybrids with various heterocycles (triazole, tetrazoles, thiadiazole, pyrimidine, and oxadiazoles) attached via a thioethanone linker group to the nitrogen-1 of the pyrazoline ring [159]. Most of them proved to be inactive or having very low cytotoxic activity against the tested cell lines, namely AsPC-1 human pancreatic adenocarcinoma, U87, and U251 glioblastoma cell lines. Compound **77** (Figure 26) exhibited promising activity with IC_50_ values of 16.9 and 11.9 μM against AsPC-1 and U251 cell lines, while it did not show any significant activity against U87. Interestingly, the DNA-cleaving efficiency of **77** proved to be much greater than cisplatin. According to the authors, it disintegrated pUC 19 DNA and this result may suggest the relationship between the DNA cleavage and the cell death.

### 5.7. Steroidal Pyrazoline Hybrids

Steroids comprise a class of natural lipids occurring in animals, plants, and fungi. They exhibit a broad range of biological activities, since, due to their conformation and lipophilicity are able to penetrate cells and bind to nucleus and membrane receptors. As anticancer agents they have been documented as enzyme inhibitors such as aromatase and sulfatase inhibitors for breast cancer, 5α-reductase for prostatic hyperplasia and even as receptor modulators such as selective estrogen receptor modulators (SERMs) or selective androgen receptor modulators (SARMs) [160]. Anticancer hybrid steroidal molecules have attracted research interest as these molecules may combine their anticancer effect with these of the heterocyclic component in a single structure. A vast number of steroidal pyrazoline conjugates have been prepared aiming for various biological activities [13], however in this review we will demonstrate the structures and biological activities of the cytotoxic ones.

**Figure 27:**
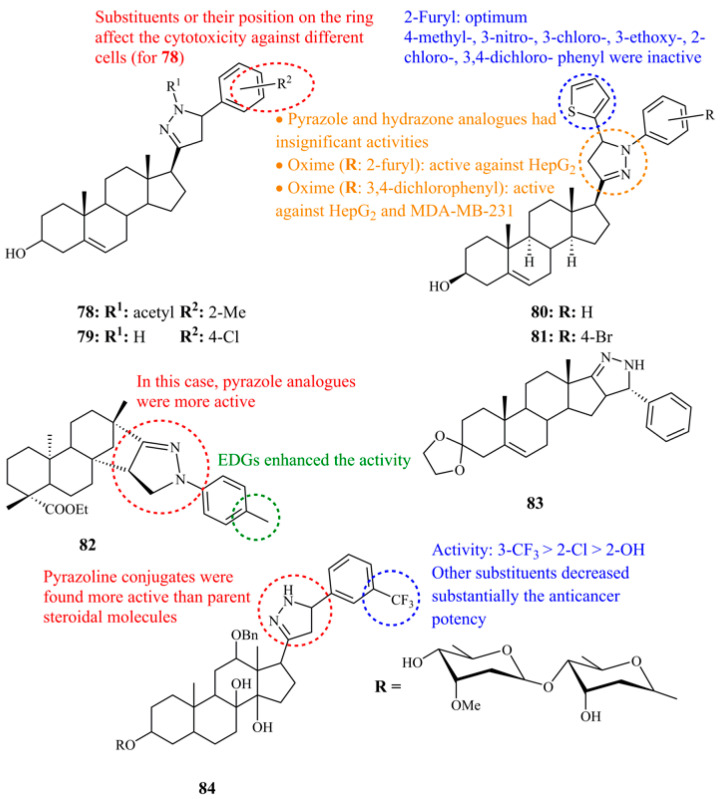
Chemical structures of anticancer D-ring substituted or fused steroidal pyrazoline hybrids.

Most steroidal conjugates involve the attachment of the pyrazoline moiety on the D-ring (the five membered cyclopentane ring) of the steroidal framework or formation of a new pyrazoline fifth ring fused on the D-ring. Compounds derived from pregnenolone were the first approach to this family of hybrid molecules. Banday et al. performed in vitro screening of the synthesized molecules and compound **78** was identified as one of the most active ones (IC_50_ values of 0.34, 0.62, 0.41, 069, 0.64, 0.79, and 1.00 μM against HC-29, HCT-15 502713, HOP-62, A549, MCF-7, and SF-295 cancer cell lines, respectively). The other members of this library involve various substituents (R—see Figure 27) like fluoride, methoxy, and chloride [161].

After having studied various steroid hybrids with heterocycles, including pyrazoles [162] as inhibitors of 17α-hydroxylase/C_17,20_-lyase, Schneider and co-workers reported the synthesis of pregnenolone-pyrazoline conjugates as potential anticancer agents [163]. Interestingly, they mentioned formation of a mixture of two steroidal epimers related to carbon-5 of the pyrazoline ring. These were found to display different biological activities and were able to be separated in their acylated form. For example, R-isomer of the compound **79**, which is one of the most potent, showed growth inhibition equal to 50.3% and 69.3% against MCF-7 and A2780 at 10 μM whereas the S-isomer displayed 25.8% and 52.3%, respectively.

To further evaluate the potential of pyrazoline as well as of other heterocyclic pregnenolone derivatives, Choudhary et al. synthesized and performed extensive screening of pregnenolone hybrids, including pyrazolines, hydrazones, pyrazoles, oximes, and their chalcone precursors against HepG_2_ and MDA-MB-230 cell lines [164]. Pregnenolone itself showed low cytotoxicity against HepG_2_ (IC_50_ = 28.97 μM) and no cytotoxity against MDA-MB-230. Among the 58 pyrazoline derivatives tested (197 compounds in total), compound **80** (Figure 27) was found highly potent against MDA-MB-230 with IC_50_ value of 0.91 μM. It was the most active compound of all for this cell line. Concerning the HepG_2_ cell line, the pyrazoline **81** (Figure 27) having a 4-bromide substituent on nitrogen-1 phenyl ring, keeping the 2-furyl group at C-5, was the most active displaying an IC_50_ value of 6.63 μM. However, for this cell line, **81** was not the most active among the compounds tested, as its chalcone precursor reached IC_50_ = 0.74 μM.

Isosteviol-fused pyrazoline **82** (Figure 27) showed the highest activity of all pyrazolines tested against Raji cell lines (IC_50_ = 3.91 μM) while having low cytotoxicity against SGC-7901, A549, and HeLa. In general, all pyrazolines demonstrated lower activities than those of the pyrazole analogues, indicating that the pyrazole fragment may play an important role in the cytotoxic activities of these conjugates [165]. The same trend followed the D-fused pyrazoline conjugates derived from androstenedione since the most active of the series, **83** (Figure 27) showed low activity against Eca-109, 446, and AGS cells with IC_50_ values of 17.5, 25.3, and 27.4 μM [166].

Kankala et al. performed the synthesis and anticancer evaluation of a series of steroidal pyrazoline conjugates derived from a steroidal glycoside extract of *Caralluma gracillis*. They investigated, as well, the role of the substituents on the phenyl ring attached at the C-5 of the pyrazoline unit. Among the 11 structures, compound **84** (Figure 27), having a *meta*-trifluoromethyl group showed the most promising activities (IC_50_ values of 2.50, 2.13, 0.34, 0.26, and 0.15 μM for A-549, MCF-7, SF-295, HCT-15, and OVACR-3 cancer cell lines, respectively) followed by 2-chloride and 2-hydroxyl [167].

**Figure 28:**
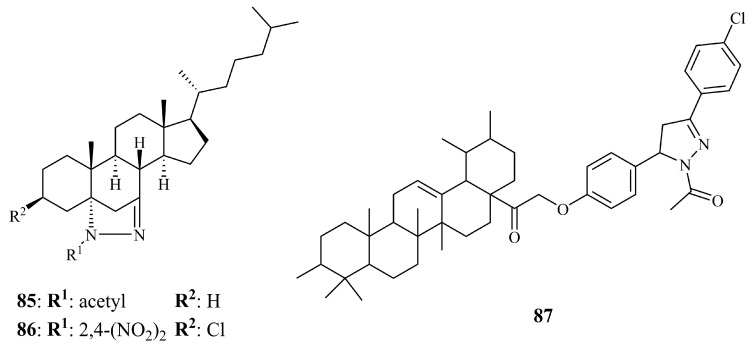
Chemical structures of anticancer B-ring fused steroidal and ursolate pyrazoline hybrids.

Shamsuzzaman et al. investigated the anticancer potential of B-ring fused steroidal pyrazoline conjugates. Derived from cholest-5-en-7-one, these derivatives were found, in principle, non-toxic to normal cells (IC_50_ > 50 μM against PBMC) [168]. Compound **85** (Figure 28) was identified as the most potent against Jurkat cell line (IC_50_ = 10.6 μM) and one of the most active against HeLa (IC50 = 22.5 μM). Following these findings, they performed a second screening on three more derivatives [169], concluding that compound **86** (Figure 28) was the most active (IC_50_ values of 15.39 and 18.31 μM for HL60 and A549, respectively).

Finally, anticancer evaluation of pyrazoline and chalcone derivatives of ursulic acid against A549, SKOV3, and HepG_2_ cancer cell lines revealed lower activities than the parent molecule [170]. Ursulic acid is a steroid-like pentacyclic triterpene with low to moderate antitumor activity (IC_50_ values of 6.3, 16.4, and 21.3 μg/mL against HepG_2_, A549, and SKOV3, respectively). From the pyrazoline derivatives, only compound **87** (Figure 28) showed slightly better cytotoxicity against the cancer cell line SKOV3 (IC_50_ = 18.9 μg/mL). Interestingly, the chalcone precursors were more potent, but none of them reached higher activity than ursulic acid against HepG_2_ cells.

### 5.8. Other Non-Classified Pyrazoline Based Conjugates

**Figure 29:**
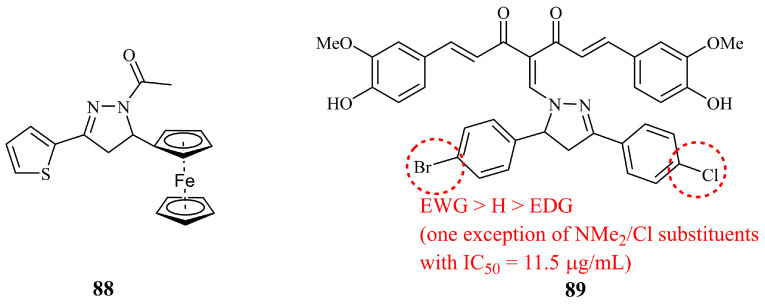
Chemical structures of other non-classified pyrazoline hybrids.

Ferrocene is a useful template for the development of anticancer agents. After the development of ferrocifen, a ferrocene-phenol hybrid, under pre-clinical trial, having a different mode of action compared to cisplatin, many researchers are designing and studying ferrocene hybrids with various other moieties including heterocycles [171]. Though many research articles have been published regarding the anticancer activities of ferrocene derivatives containing pyrazolyl-moiety [172,173,174], there is only one on pyrazoline hybrids. Compound **88** (Figure 29) was found effective at growth inhibition on cancer colonies and, moreover, non-toxic [175].

Meanwhile, in a very recent publication, Chaudhary et al. reported the synthesis and biological evaluation of curcumin-pyrazoline condensates as anticancer agents against human cervical cancer HeLa [176]. Compound **89** exhibited 5-fold better cytotoxicity in comparison to curcumin (IC_50_ values of 8.7 and 42.4 μg/mL for **89** and curcumin, respectively). In addition, *in silico* studies pointed out that **89** demonstrates good binding interactions with the human IKK-β protein.

## 6. Conclusions

The idea of designing hybrid molecules consisting of two or more therapeutic units in a single molecule is generally new. The strategy of molecular hybridization has been employed by medicinal chemists to develop novel bioactive compounds that aim towards multiple targets, being at the same time less vulnerable to drug resistance. Pyrazolines are versatile molecules with potent anticancer activities, thus they have been selected by many researchers for the development of such structures.

In this review, we presented a variety of literature reports on anticancer pyrazoline hybrids containing an additional heterocyclic or non-heterocyclic framework. Overall, the structures described were more potent than their parent compounds.

Alongside, we tried to shed light onto structure activity relationships wherever it was possible and for the compounds having at least moderate activities. 1-Pyrazolines and 3-pyrazolines are certainly underrepresented and have not been investigated adequately. However, even for 2-pyrazolines, since this field comprises of relatively recent works, there are not many comprehensive structure activity relationship studies for many cancer cell lines, protein targets, toxicology, and in vivo tests.

Among the hundreds of structures, one can identify some common modalities; groups, atoms, and structural conformations that favor the anticancer activities. As a general trend, *p*-methoxyphenyl groups at C-3 and C-5 of the ring usually enhance cytotoxicity, as does, in many cases, the trimethoxyphenyl unit inspired from combretastatin A4. In parallel, in other studies, electron withdrawing groups, mostly fluoride or chloride increased the activity. Coumarin, quinoline, indole, and thiazole components have been found as very potent. Overall, there is not a generalized SAR rule for enhancing the cytotoxicity and it is heavily dependent on the structure configuration or functionalization of the conjugate molecule.

To conclude, pyrazoline is an important five membered nitrogen heterocycle. The ring is quite stable and has inspired chemists to carry out various structural modifications to enhance activity, selectivity, and/or pharmacological properties. This has propelled the development of distinct pyrazolines towards the area of molecular hybridization to achieve maximum multimodal activity, as it has been highlighted by the content of this review. Since this topic is relatively new, it is anticipated that a lot of frontier research will be performed, and we believe that scientists working on such derivatives will find valuable information to further advance the structural diversity and anticancer applications of novel pyrazoline-based hybrid drugs.
